# Supplementary material for: Bioluminescence Production by Turnip Yellows Virus Infectious Clones: A New Way to Monitor Plant Virus Infection
Source: Int J Mol Sci. 2022 Nov 8;23(22):13685. doi: 10.3390/ijms232213685 (PMC9692398; doi:10.3390/ijms232213685)
Supplement: Supplementary file 1 [file ijms-23-13685-s001.zip › Table-S1.pdf]

Table S1: Primers used in the experiments.

Primers	5' -> 3' Sequence
FP3	GCTGTGTTAGACGGTCGCATGGTCTTCACACTCGAAG
RP1	GGTCAGGTTGTATCCCTTCATTCATACGGGATGATGACATG
FP2	CATGTCATCATCCCGTATGAATGAAGGGATACAACCTGACC
RP3	CTTCGAGTGTGAAGACCATGCGACCGTCTAACACAGC
FP4	GAGGCTTGCCCCTTCCTGTTATGGTCTTCACACTCGAAG
RP4	GGTCAGGTTGTATCCCTTCATTACGCCAGAATGCGTTCG
FP5	CGAACGCATTCTGGCGTAATGAAGGGATACAACCTGACC
RP5	CTTCGAGTGTGAAGACCATAACAGGAAGGGGCAAGCCTC
221	ATTGTCGACACCGAAGTGCCGTA
946	ATGGTCTTCACACTCGAAGA
947	CGCCAGAATGCGTTCGCACA
1048	GGTCTGAGCGGCGAC
Luc-For	***ctaTCTAG ATG GGG GGT CTG AGC GGC GAC***<br>*XbaI M G G L S G D*
Luc-Rev	***AGTGAGCTCACAGATCCTCTTCGGATATCAGCTTTTGTTCGCTGCCGCCGCCCAGCGCCAGAATGCG***<br>*SacI myc tag linker C66Luc end*

Underlined sequences are complementary to NanoLuciferase sequence.
