# Supplementary material for: Bioluminescence Production by Turnip Yellows Virus Infectious Clones: A New Way to Monitor Plant Virus Infection
Source: Int J Mol Sci. 2022 Nov 8;23(22):13685. doi: 10.3390/ijms232213685 (PMC9692398; doi:10.3390/ijms232213685)
Supplement: Supplementary file 1 [file ijms-23-13685-s001.zip › Figure-S1.pdf]

Supplementary Figure S1: Characterization of *N. benthamiana* 35S:C66-NL plants

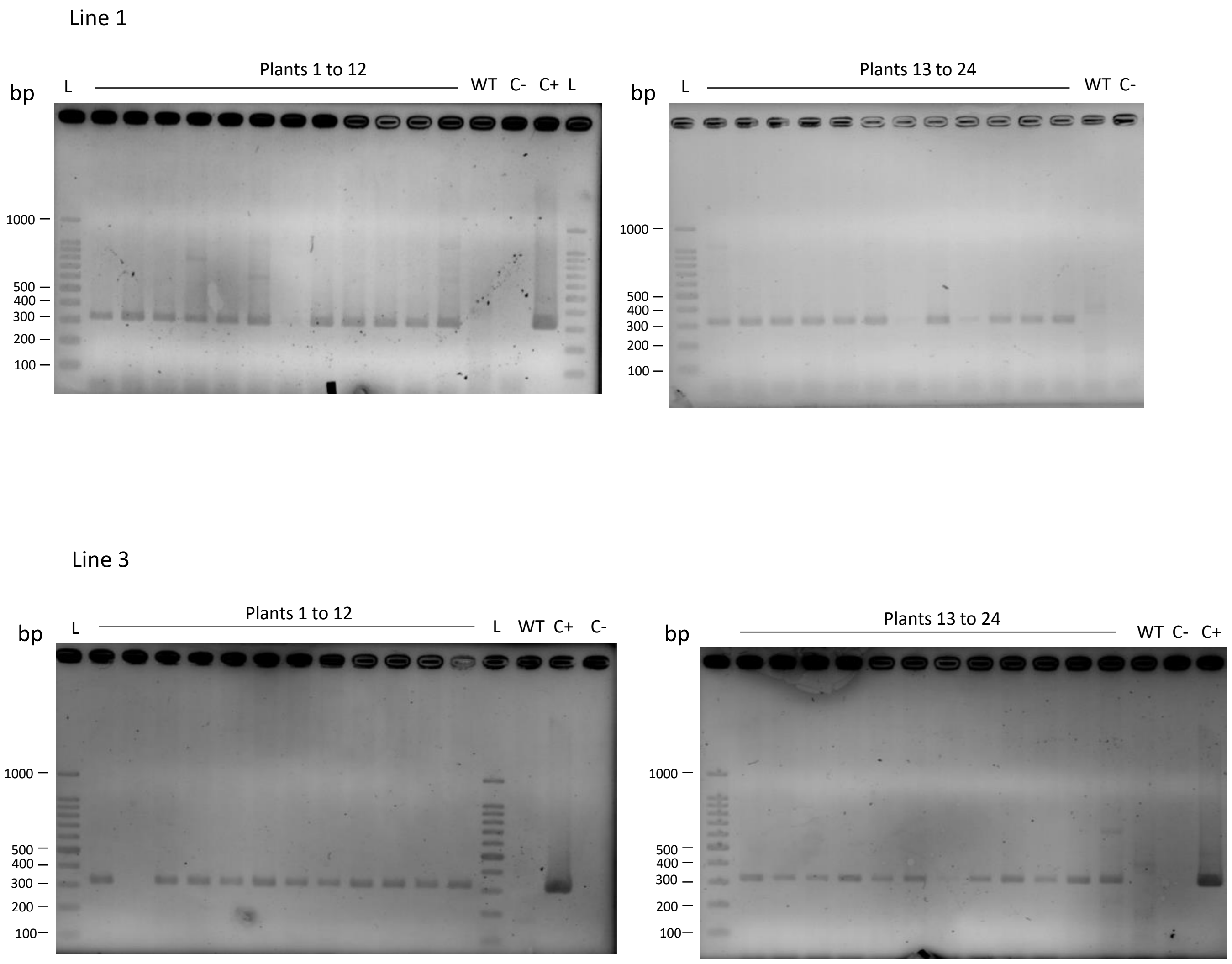

Figure S1. Detection by PCR of the C66-NL sequence in24 *Nicotiana benthamiana* transgenic plants (Lines 1 and 3) before agro-inoculation with TuYV-N65-NL. PCR was performed directly on plant tissue (1 mm²) with Kapa3G plant PCR kit according to the provider's instructions (CliniSciences, Nanterre, France). PCR was carried out with forward primer 5'-GGTCTGAGCGGCGAC-3' and reverse primer 5'-CGCCAGAATGCGTTCGCACA-3' amplifying a 317 bp fragment of the C66-NL sequence.

WT : *Nicotiana benthamiana* wild type plant

C- : Negative control (H_2O)

C+ : Positive control (plasmid used for *N. benthamiana* transformation)
